# Endoscopic bile duct stenting as preliminary treatment of complicated choledochal malformation before definitive surgery

**DOI:** 10.1002/jpn3.70260

**Published:** 2025-11-06

**Authors:** Johannes Weidner, Joachim Friedrich Kuebler, Andrea Schneider, Jens Dingemann, Ulrich Baumann, Eva‐Doreen Pfister, Nagoud Schukfeh

**Affiliations:** ^1^ Department of Pediatric and Adolescent Surgery Hannover Medical School Hannover Germany; ^2^ Department of Gastroenterology, Hepatology, Infectious Diseases and Endocrinology Hannover Medical School Hannover Germany; ^3^ Department of Pediatric Kidney, Liver, and Metabolic Diseases, Division of Pediatric Gastroenterology and Hepatology Hannover Medical School Hannover Germany

**Keywords:** endoscopic retrograde cholangiopancreatography, hepatobiliary, liver, pediatric

## Abstract

**Objectives:**

Acute pancreatitis or obstruction is considered an indication for urgent surgery in patients with choledochal malformation. However, in this inflammatory stage, surgery can be challenging. Our approach is to treat the acute obstruction by endoscopic retrograde cholangiopancreatography (ERCP), papillotomy, and placement of a bile duct stent. Subsequently, surgical resection of choledochal malformation is performed after the inflammation has subsided.

**Methods:**

In this retrospective study, we included all patients who underwent surgical resection in our department for choledochal malformation with prior ERCP between 12/2013 and 12/2024. Post‐ERCP data of patients who received additional stenting were analyzed.

**Results:**

Twenty patients (75% female) with a complicated choledochal malformation and a median age of 5.6 years (range 0.4–18.3 years) were included. Fourteen patients presented with biliary pancreatitis, six of these with an additional symptomatic cholelithiasis. Twelve patients with complicated choledochal malformation underwent papillotomy and stenting of the common bile duct with post‐interventional clinical improvement. Out of these, three underwent a second and two patients a second and a third ERCP with stent replacement before surgery. One patient received six ECRPs in another hospital before surgery. After a median interval of 9 weeks after stenting (range, 2–34 weeks), three patients (25%) received open and nine (75%) laparoscopic resections of the choledochal malformation, with two conversions to open surgery (22%) due to massive adhesions. Surgery was uneventful in all patients.

**Conclusion:**

Our data suggest that preoperative ERCP and stenting of the common bile duct is a valid option for patients with choledochal malformation complicated by pancreatitis or obstruction, making a laparoscopic approach feasible in the majority of patients.

## Abstract (highlights)

**What is Known:**

- Choledochal malformation complicated by acute pancreatitis or obstruction is an indication for urgent surgery.
- Endoscopic retrograde cholangiopancreatography (ERCP) is a common diagnostic tool for choledochal malformation.
- Timing of surgery remains controversial.

**What is New:**

- ERCP and bile duct stenting are a feasible first‐line treatment option in the acute phase of obstruction in patients with choledochal malformation.
- Laparoscopic surgery can be postponed safely after ERCP with low rates of complications.

## Graphical abstract

Left picture: ERCP before stent placement.

Right picture: ERCP after placement of two stents.

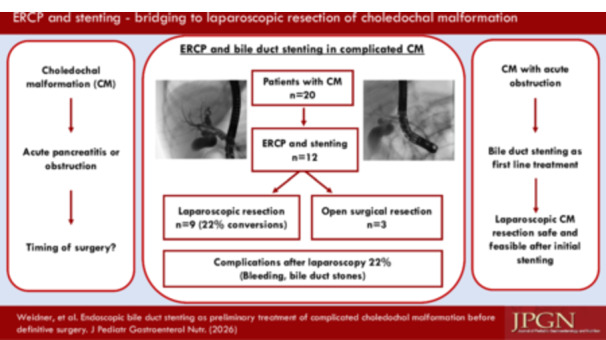

## 1 INTRODUCTION

Choledochal malformation is a rare condition that, in most cases, presents in the first decade of life. Females are more often affected than males (3–4: 1).^1^ Reports on incidence range from 1:1000 live births in Asian countries to a recently estimated incidence of 1:37,400 in Scandinavian populations.^1^, ^2^, ^3^ Most often, classification of choledochal malformations follows the Todani modification of the Alonso‐Lej classification with type I being the most common type (50%–80%).^4^

Although some cases are diagnosed prenatally and operated on at a young age before developing clinical symptoms, most children present with abdominal pain, vomiting, fever, and jaundice at a late stage.^5^, ^6^ In cases of distal obstruction, patients may present with a combination of fever, cholangitis, or pancreatitis.

Conservative treatment may lead to clinical improvement. However, acute pancreatitis or obstruction due to choledocholithiasis is considered an indication for urgent surgery in patients with choledochal malformation.^7^, ^8^ In this inflammatory stage, surgery can be challenging. To conquer increased rates of complications, authors suggest a 6‐week interval between the last episode of pancreatitis and surgery.^9^

Endoscopic retrograde cholangiopancreatography (ERCP) became increasingly important in diagnosing and treating choledochal malformations, providing the possibility of immediate endoscopic papillotomy and bile duct stenting. ERCP has been found to be a safe and reliable procedure for children with choledochal cysts remaining a reasonable addition or alternative to radiologic approaches.^10^, ^11^, ^12^

Previously published data indicate that the majority of patients (up to 86%) undergo loss to follow‐up after resection of a choledochal malformation, with follow‐up rates decreasing over time. The reported median follow‐up time ranged from 4 to 9 years.^13^, ^14^, ^15^, ^16^

There still is no consensus today about how and when to treat patients with complicated choledochal malformation.^17^ We present our approach of treating the acute obstruction by ERCP, papillotomy, and placement of a bile duct stent. Elective surgical choledochal malformation resection can be safely performed after the inflammation has subsided. The aim of our study is to evaluate the advantage of preoperative ERCP in cases of children with choledochal malformations complicated by biliary pancreatitis over the primary surgical approach.

## 2 METHODS

### 2.1 Ethics statement

The study was approved by the ethics committee of the Hannover Medical School (number 8230_BO_K_2018).

### 2.2 Study design and participants

We included all patients who received surgical resection of a choledochal malformation in our institution with prior ERCP between 12/2013 and 12/2024. The medical records of all patients were retrospectively analyzed. Pre‐ and postoperative clinical and laboratory data were obtained for all patients who underwent additional stenting of the common bile duct. Data are given in median and range unless stated otherwise. ERCPs in our hospital were exclusively performed by Andrea Schneider.

### 2.3 Diagnostics

Pancreatitis was diagnosed when abdominal pain was associated with elevated serum levels of pancreatic lipase of >180 U/l and/or elevated serum levels of α‐amylase of >300 U/l. In case of suspicion of pancreatic or biliary stones, magnetic resonance cholangiopancreatography (MRCP) was performed. A common channel was defined as the abnormal junction of the pancreatic duct and common bile duct that occurs outside the duodenal wall to form a long common channel (>8 mm).

### 2.4 ERCP, papillotomy, and stenting

Indication for ERCP was a severe clinical course with biliary obstruction or pancreatitis as well as unclear imaging results. ERCP was performed under general anesthesia. In our hospital a video duodenoscope with an outer diameter of 7.5 mm and a 2‐mm instrumentation channel (PJF‐160 Olympus) was utilized in patients <1 year, in patients >1 year a video duodenoscope with an outer diameter of 11 mm and a 3.2‐mm instrumentation channel (JF‐140R Olympus) was utilized, both used insufflation of CO_2_. Papillotomy was routinely performed. If there was no sufficient bile flow after papillotomy, stent placement was carried out using a 5 French 5 cm Flexima Biliary Stent (Boston Scientific, Spencer, USA) in patients <1 year. In patients >1 year 7 French or 8.5 French 5 cm Flexima Biliary Stents (Boston Scientific, Spencer, USA) were primarily used. Antibiotic prophylaxis (cefotaxime 35 mg/kg per dose every 8 h) was administered intravenously for at least 24 h. Our concept included ERCP as a bridging therapy until definite surgery was performed to achieve optimal conditions for resection of choledochal malformation.

### 2.5 Resection of choledochal malformation

The technique of laparoscopic resection of choledochal malformation has been described previously including end‐to‐side hepaticojejunostomy.^18^ Open surgical resection of the choledochal malformation was carried out according to the standard technique.^19^ Standard approach for resection of choledochal malformation was laparoscopy. Indication for open surgery was prior abdominal surgery with expected massive adhesions.

### 2.6 Follow‐up

Our postoperative follow‐up concept includes yearly follow‐up including abdominal ultrasound and control of laboratory parameters. All patients are referred to a structured transition program during adolescence and yearly lifelong clinical, sonographic and laboratory examinations including serum CA19‐9 and CEA levels are recommended.^20^

## 3 RESULTS

### 3.1 Patients' characteristics

During the study period, 55 patients underwent surgical treatment of a choledochal malformation in our department. Twenty of them (75% female) with a median age of 5.6 years (range 0.4–18.3 years) received a preoperative ERCP (Table 1). Twelve patients received a biliary stent. All of these 12 patients initially presented with biliary pancreatitis, and an additional symptomatic cholelithiasis in six patients (50%). Four patients (30%) had received ERCP and stenting in other hospitals (patient no. 4, 6, 9, and no. 11). Two patients (17%) had undergone laparoscopic cholecystectomy in other hospitals 2 and 22 months before surgery (patient no. 4 and no. 9), respectively. Patient no. 8 had undergone heminephrectomy of the left upper pole due to a nephroblastoma 3 months before surgery (Table 2).

**Table 1:** Patient characteristics.

Patients' number	Age (Years)	Gender	MRCP	Todani	Cholelithiasis	Stent
1	1.5	F	Yes	4a	Yes	Yes
2	2.8	F	No	1	No	Yes
3	2.5	F	No	1	No	Yes
4	5.7	F	Yes	4b	Yes	Yes
5	2.1	F	Yes	4a	No	Yes
6	18.3	M	Yes	1	No	Yes
7	5.8	M	Yes	1	Yes	Yes
8	2.2	M	No	1	No	Yes
9	9.6	F	Yes	1	Yes	Yes
10	9.5	F	Yes	1	No	Yes
11	7.6	F	Yes	1	Yes	Yes
12	8.1	F	Yes	4a	Yes	Yes
13	2.3	F	Yes	1	No	No
14	0.4	M	No	1	No	No
15	1.7	F	Yes	1	No	No
16	12.5	F	Yes	1	No	No
17	5.4	M	Tes	4a	Yes	No
18	9.4	F	Yes	1	No	No
19	15.3	F	Yes	1	No	No
20	3.1	M	Yes	1	Yes	No

*Note*: Todani type based on Todani classification.^4^

Abbreviation: MRCP, magnetic resonance cholangiopancreatography.

**Table 2:** Procedure details in stented patients.

Patients' number	ERCPs (*n*)	Papillotomies (*n*)	ERCP‐OP (weeks)	Surgery type	OP time (min)
**1**	3	2	17	MIS	283
2	1	1	3	MIS	218
3	1	1	2	MIS	290
4	2	1	34	Converted	297
5	1	1	11	Open	154
6	1	1	4	MIS	545
7	1	1	4	MIS	324
8	2	1	16	Open	174
9	6	0	7	Open	504
10	3	1	6	MIS	552
11	1	1	19	Converted	579
12	2	1	29	MIS	541

*Note*: ERCP‐OP: time between last ERCP and surgery in weeks.

Abbrviations: Converted, conversion from laparoscopy to open surgery; ERCP, endoscopic retrograde cholangiopancreatography; MIS, minimally invasive surgery.

### 3.2 ERCP and stenting

ERCP was successfully performed, including papillotomy and stenting of the common bile duct, leading to long‐term clinical and biochemical improvement in all patients. Median total bilirubin levels decreased from 15 µmol/l (range 3–199 µmol/l) before ERCP to 3 µmol/l (range 0–19 µmol/l) at the time of surgery. None of the patients had clinical or biochemical signs of pancreatitis at the time of surgery.

#### 3.2.1 Early complications (≤30 days) after ERCP

Early complications occurred in five patients (25%). A peri‐interventional bile leak was observed in patient no. 1 following papillotomy which was effectively managed through conservative measures. Stent placement was deferred and subsequently performed during a second ERCP. This patient received 3 ERCPs in total before surgery. Two out of 12 patients (17%) developed postinterventional symptoms including abdominal pain (patient no. 3) and subfebrile temperature (patient no. 8) after stent placement, which were managed conservatively in both cases. After ERCP and papillotomy without stenting 2 out of 8 patients (25%) developed a post‐ERCP pancreatitis managed conservatively.

#### 3.2.2 Late complications (>30 days) after ERCP

**Figure 1:**
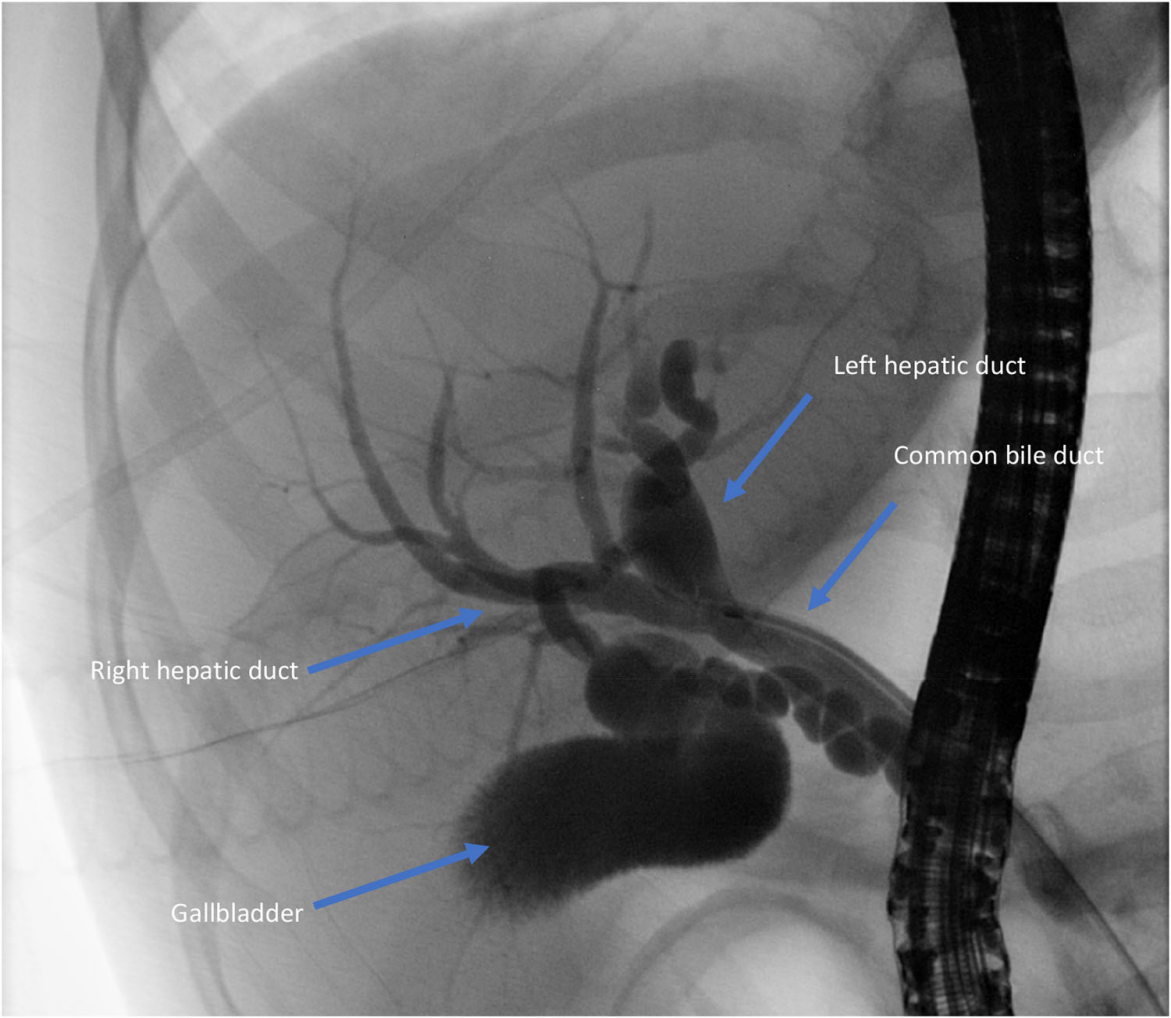
Endoscopic retrograde cholangiopancreatography before stent placement in patient 3. Choledochal cyst class 4a based on Todani classification.^4^

**Figure 2:**
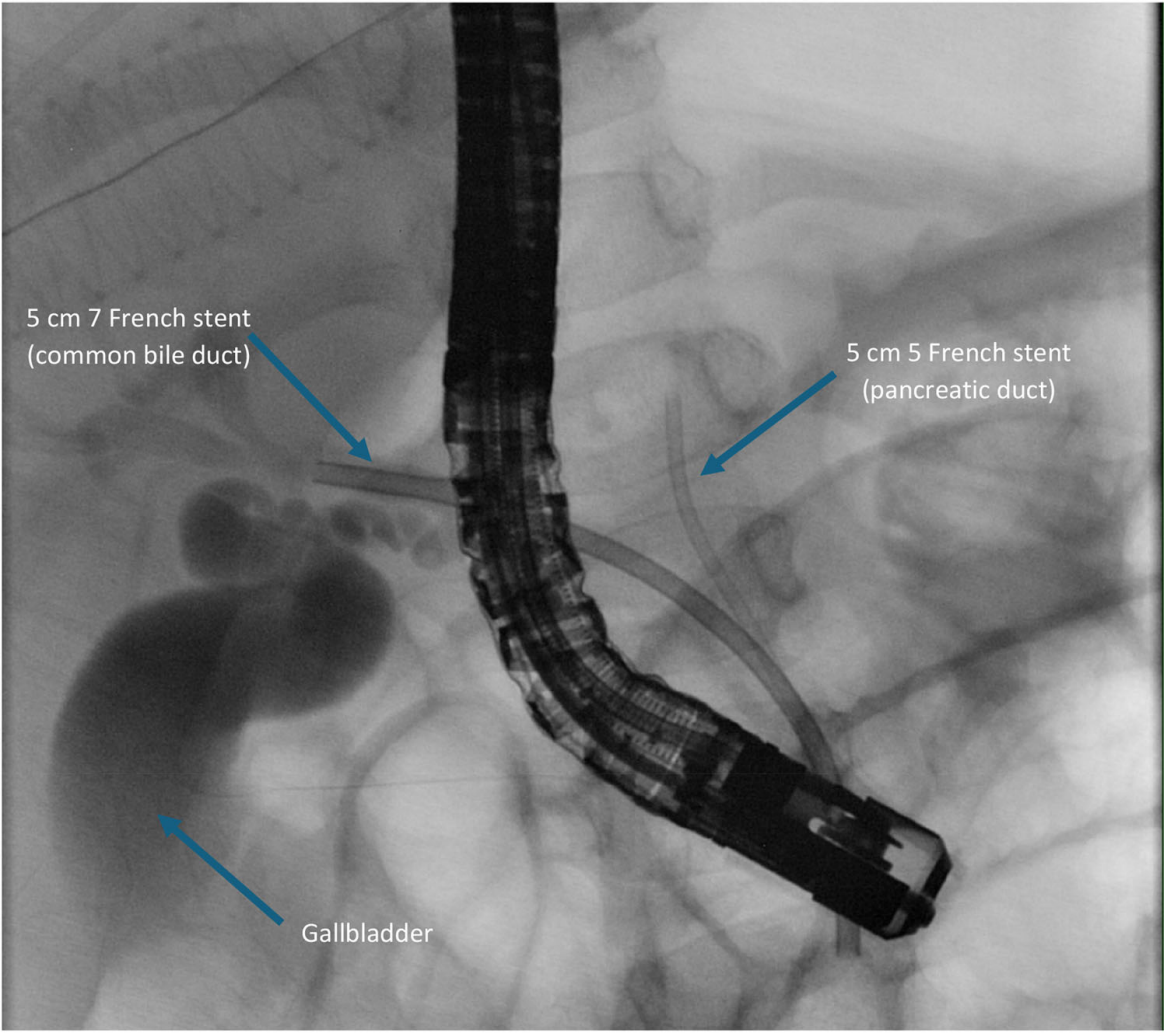
Endoscopic retrograde cholangiopancreatography after placement of a 5 French 5 cm stent in pancreatic duct and 7 French 5 cm stent in common bile duct in patient 3. No contrast enhancement of the choledochal malformation after drainage.

Late complications more than 30 days after ERCP occurred in three patients (25%). These patients underwent a second ERCP due to stent dislocation. In patient no. 10, a delayed diagnosis of choledochal malformation led to three ERCPs being performed before surgery. Patient no. 9 underwent 6 ERCP procedures, including elective stent replacements, at another institution before referral to our hospital (Figures 1 and 2).

### 3.3 Surgical resection of choledochal malformation after ERCP and stenting

After a median interval of 9 weeks (range, 2–34 weeks), three patients (25%) underwent conventional open and nine patients (75%) underwent laparoscopic resection of the choledochal malformation, with two conversions to open surgery (22%). Indications for conversion included massive adhesions following prior hepatobiliary surgery (patient no. 2) and recurrent episodes of pancreatitis (patient no. 11). Surgery was uneventful in all patients. Median operation time was 324 min (range 218–552 min) for the laparoscopic and 174 min (range 154–504 min) for the open procedures.

### 3.4 Follow‐up and complications after surgical resection

Postoperative follow‐up included a clinical examination at the tending pediatrician 1 week after discharge and after that yearly clinical follow‐up including abdominal ultrasound examination, control of laboratory parameters of cholestasis as well as of CA19‐9 and CEA beginning at the age of 18 at our outpatient clinic or at a pediatric gastroenterologist as has been recommended before.^20^ The median follow‐up duration was 86 weeks (range: 3–493 weeks), with a follow‐up rate of 83%.

Postoperative complications included one case of postoperative bleeding on the day of surgery treated by operative revision and hemostasis (patient no. 12) and one case of postoperative intrahepatic cholelithiasis occurring >30 days after surgery treated by repeated ERCPs and stenting (patient no. 2).

## 4 DISCUSSION

Our data confirm that preoperative ERCP and stenting of the bile duct is feasible as a bridging procedure before resection of choledochal malformation in children. In most patients, subsequent laparoscopic choledochal malformation resection in the interval was feasible and uneventful.

Recently, we published our results on all laparoscopic choledochal malformation resections in our department and reported a conversion rate of 30%.^21^ In the present collective of patients who underwent ERCP before laparoscopic choledochal malformation resection, our conversion rate was 22%. Possibly, the prior ERCP and stenting of the bile duct leads to clinical improvement and decrease of the intraperitoneal inflammation which may facilitate the laparoscopic preparation of the choledochal malformation. Due to the small sample size, the difference did not reach statistical significance.

In another recent study published by our group, samples of resected choledochal malformation from 42 pediatric patients were analyzed histologically and mutational analysis of the protooncogenes kirsten rat sarcoma virus (KRAS) and B‐rapidly accelerated fibrosarcoma (BRAF) as early markers of malignant alteration in cholangiocytes was performed showing frequent chronic inflammatory changes in choledochal malformation of infants and children. However, the lack of KRAS and BRAF mutations suggested that no malignant changes have been initiated in this group of patients undergoing early resection. Despite the absence of malignant changes in biopsy specimen, we continue to recommend timely resection of choledochal malformations.

In one patient, operation time of laparoscopic choledochal malformation resection was particularly long. This patient initially presented with a clinically distinct pancreatitis and underwent laparoscopic choledochal malformation resection only 4 weeks after ERCP and stenting. In this patient, laparoscopic preparation of the choledochal malformation was hindered by a still ongoing peritoneal inflammation. This goes along with evidence from the surgical management of other inflammatory diseases in adults indicating that delayed intervention may be associated with lower conversion rates, decreased operative duration, and reduced length of hospital stay.^22^ We therefore now recommend a longer interval of 6 weeks following ERCP and stenting before laparoscopic choledochal malformation resection is performed.

There have been different approaches as bridging procedures for choledochal malformation complicated by perforation. Kang et al. reported in 2008 on an adult patient with perforated choledochal malformation, which was managed with preoperative percutaneous transhepatic cyst drainage, followed by definite surgery for choledochal cyst, cyst excision, and reconstruction with Roux‐en‐y hepaticojejunostomy.^23^ The authors concluded that preoperative percutaneous cyst drainage seemed to be useful and effective in managing complicated choledochal cysts and stated that this procedure garners time for patients' general conditionand laboratory findings to improve to the extent that general surgery can be performed.^23^ Later, in 2015 Minagawa reported on a similar case of a woman with perforated choledochal malformation who underwent endoscopic nasal biliary drainage as a bridging procedure. The drainage was placed by transnasal endoscopy and consecutive ERCP leading to clinical improvement. After an interval of 22 days choledochal malformation resection was performed via laparotomy.^24^ The authors reported that the patient underwent subtotal stomach‐preserving pancreatoduodenectomy because of massive inflammation around the pancreas head which was too severe to perform cyst excision safely.

Both above‐mentioned reports considered adult patients, whereas Song et al. published a comparative study on three methods of minimally invasive external drainage in pediatric patients with complicated choledochal malformation.^25^ The retrospective study included 20 children who underwent biliary drainage before their definitive surgery due to acute symptomatic choledochal malformation. Among them, six underwent ultrasound‐guided percutaneous biliary drainage, eight underwent laparoscopic bile duct drainage, and six underwent laparoscopic cholecystostomy. The authors found that preoperative abdominal pain, fever, and jaundice symptoms were effectively relieved by the three approaches. They also found no significant differences in terms of the recovery of liver functions. The authors favored the laparoscopic cholecystostomy for the majority of patients due to its simple technique, stable fixation, least influence on the common bile duct, and benefit of definitive surgery.

In another retrospective study, Drabek et al. reported on their experience with ERCP in children with choledochal malformation and/or abnormal pancreatobiliary junction.^10^ The group evaluated ERCPs performed to diagnose abnormalities of the pancreaticobiliary junction, mainly choledochal cysts, in 112 children. In one‐third of the children, they performed endoscopic papilla sphincterotomy, and endoscopic drainage in 55%. The authors stated that ERCP would be a reasonable alternative for the diagnosis of choledochal cysts and/or abnormalities of the pancreaticobiliary junction because MRCP still has limited ability to diagnose anomalies such as the common channel. They stated that in addition, ERCP would provide therapeutic endoscopic modalities.^10^ However, this group performed solely ERCP without leaving some kind of biliary drainage.

In a recently published study, Rollo et al. reported on 31 pediatric patients with congenital choledochal malformation receiving either preoperative ERCP and biliary lavage (26%) or ERCP and sphincterotomy (74%) for the treatment of acute pancreatitis with or without choledocholithiasis. In both groups time to surgery was approximately 4 months. In that study, patients underwent open resection of the malformation, which is in contrast to our laparoscopic approach.^26^

In contrast, Sun reported on 17 children with congenital biliary dilatation, who underwent ERCP with five patients receiving a nasobiliary drainage and 12 undergoing biliary drainage through double pigtail tubes.^27^ All patients in that study achieved successful biliary drainage. Postoperative pancreatitis occurred in one patient. There were no other major complications related to ERCP. Open or laparoscopic surgery for congenital biliary dilatation were performed 2 weeks to 2 months after endoscopic biliary drainage when the pigtail tube was taken out simultaneously. No complications such as bile leakage or cholangitis were described. The authors concluded that endoscopic biliary drainage is a safe technique that could be used to resolve congenital biliary dilatation‐associated cholangitis, to evaluate the biliary tract.^27^

In a recent publication, Zhang et al. discuss preoperative ERCP and papillotomy in a pediatric population to prevent preoperative complications in patients with choledochal malformations. Similar to our approach, namely stenting of the bile duct, endoscopic nasobiliary drainage was performed in 94% of these patients. The median time interval between ERCP and surgery was 11 days, mean duration of surgery was 270 min. Seventy‐two percent of patients received laparoscopic surgery, 10% developed complications which were conservatively managed.^28^ In line with our results, these data show the feasibility of permanent biliary drainage until definitive surgery. However, based on above‐mentioned data, we recommend a 6‐week interval until laparoscopic resection of the choledochal malformation making the internal biliary drainage more patient‐friendly.^9^, ^22^

One limitation of our study is that the preoperative clinical data of the patients were retrospectively collected, another one is the small number of patients. Larger studies with higher patient numbers are recommended to confirm our results.

## 5 CONCLUSION

Our data suggest that preoperative ERCP and stenting of the common bile duct is a valid option for patients with choledochal malformation complicated by pancreatitis or obstruction, possibly facilitating a laparoscopic approach in the majority of patients.

## CONFLICT OF INTEREST STATEMENT

The authors declare is no conflict of interest.
